# Unraveling Biohydrogen Production and Sugar Utilization Systems in the Electricigen *Shewanella marisflavi* BBL25

**DOI:** 10.4014/jmb.2212.12024

**Published:** 2023-02-15

**Authors:** Sang Hyun Kim, Hyun Joong Kim, Su Hyun Kim, Hee Ju Jung, Byungchan Kim, Do-Hyun Cho, Jong-Min Jeon, Jeong-Jun Yoon, Sang-Hyoun Kim, Jeong-Hoon Park, Shashi Kant Bhatia, Yung-Hun Yang

**Affiliations:** 1 Department of Biological Engineering, College of Engineering, Konkuk University, Seoul 05029, Republic of Korea; 2 Green and Sustainable Materials R&D Department, Research Institute of Clean Manufacturing System, Korea Institute of Industrial Technology (KITECH), Cheonan 31056, Republic of Korea; 3 School of Civil and Environmental Engineering, Yonsei University, Seoul 03722, Republic of Korea; 4 Sustainable Technology and Wellness R&D Group, Korea Institute of Industrial Technology (KITECH), Jeju 63243, Republic of Korea

**Keywords:** *Shewanella*, whole-genome sequencing, biohydrogen production, carbon source utilization system

## Abstract

Identification of novel, electricity-producing bacteria has garnered remarkable interest because of the various applications of electricigens in microbial fuel cell and bioelectrochemical systems. *Shewanella marisflavi* BBL25, an electricity-generating microorganism, uses various carbon sources and shows broader sugar utilization than the better-known *S. oneidensis* MR-1. To determine the sugar-utilizing genes and electricity production and transfer system in *S. marisflavi* BBL25, we performed an in-depth analysis using whole-genome sequencing. We identified various genes associated with carbon source utilization and the electron transfer system, similar to those of *S. oneidensis* MR-1. In addition, we identified genes related to hydrogen production systems in *S. marisflavi* BBL25, which were different from those in *S. oneidensis* MR-1. When we cultured *S. marisflavi* BBL25 under anaerobic conditions, the strain produced 427.58 ± 5.85 μl of biohydrogen from pyruvate and 877.43 ± 28.53 μl from xylose. As *S. oneidensis* MR-1 could not utilize glucose well, we introduced the *glk* gene from *S. marisflavi* BBL25 into *S. oneidensis* MR-1, resulting in a 117.35% increase in growth and a 17.64% increase in glucose consumption. The results of *S. marisflavi* BBL25 genome sequencing aided in the understanding of sugar utilization, electron transfer systems, and hydrogen production systems in other *Shewanella* species.

## Introduction

With the global population growing exponentially, energy demand has increased remarkably, placing unprecedented pressure on the global ecosystem. To solve this issue, various renewable energy sources and production methods are currently under research [1-3]. Among them, microbial fuel cells and bioelectrochemical systems, which are recognized as novel biotechnologies for energy generation via sustainable biodegradation of chemical compounds using microorganisms as catalysts [4, 5], have been promoted as a solution. Many electricigens, including archaea, Cyanobacteria, Firmicutes, Proteobacteria (α-Proteobacteria, β-Proteobacteria, γ-Proteobacteria, and δ-Proteobacteria), yeast, and eukaryotic algae oxidize organic compounds and transfer electrons to anodes [6-8]. Among these electricigens, *Shewanella* species are widely studied owing to their metal-reducing activity that reduces solid metal oxides [9]. The electrogenic facultative anaerobe *Shewanella oneidensis* MR-1 is a γ-Proteobacterium belonging to the order Alteromonadales [10]. *Shewanella oneidensis* MR-1 has multiple extracellular electron transport mechanisms, including direct electron transfer to electrodes via c-type cytochromes, electroactive biofilms, conductive nanowires/pili, and indirect transfer via mediators/shuttles. Based on these characteristics, this strain can reduce insoluble metal oxides and is a promising agent for bioremediating contaminated soils and water [11, 12]. Apart from these abilities, *Shewanella oneidensis* MR-1 can also accumulate formate via lactate or pyruvate oxidation and produce hydrogen [13, 14]. Although most microbial biohydrogen production occurs in *Clostridium* spp., *Enterobacter* spp., and *Bacillus* spp. [15-17], *S. oneidensis* MR-1 is also considered a potential hydrogen producer owing to its ability to oxidize carbon sources. Moreover, *S. oneidensis* MR-1 has various hydrogenases and could be engineered to produce biohydrogen [18]. Despite these advantages, some disadvantages hinder the application of *S. oneidensis* MR-1 [19]. One major disadvantage is the limited utilization of particular carbon sources, such as *N*-acetylglucosamine, lactate, and pyruvate [20, 21].

Previously, we reported that the newly isolated *S. marisflavi* BBL25 strain could utilize multiple carbon sources, such as glucose, xylose, galactose, fructose, and lactose [22, 23]. In addition, comparable amounts of electricity could be produced from lignocellulosic biomass. Thus, *S. marisflavi* BBL25 has more practical applications in hydrogen production than *S. oneidensis* MR-1 based on carbon source utilization. Through whole-genome analysis, we not only identified various carbon source-consuming pathways but also determined the associated electron transfer and hydrogen production systems. Based on these findings, we hypothesized that *S. marisflavi* BBL25 could produce hydrogen in the presence of exogenous electron acceptors using various carbon sources. Therefore, we aimed in this study to explore hydrogen production and carbon source metabolism in *S. marisflavi* BBL25 using whole-genome sequencing and demonstrate the hydrogen production system of *S. marisflavi* BBL25. Hence, we herein present the process from genetic information discovery to biohydrogen production and carbon source utilization by *S. marisflavi* BBL25.

## Materials and Methods

### Whole-Genome Sequencing and Genomic DNA Assembly and Annotation

Whole-genome sequencing of *Shewanella marisflavi* BBL25 was performed, and the results were analyzed by ChunLab Inc. (Korea) using PacBio SMRT Analysis 2.3.0 and the HGAP2 protocol (Pacific Biosciences, USA). The contigs from the PacBio sequencing data were circularized using Circlator 1.4.0 (Sanger Institute, UK). PacBio libraries were sequenced using PacBio P6C4 chemistry in an 8-well SMRT Cell v3 with the PacBio RS II system. Functional gene annotation of the whole-genome assembly was performed using the EzBioCloud genome database [24]. Coding sequences were classified by referencing orthologous groups (EggNOG 4.5; http://eggnogdb.embl.de). As a result, we obtained a 4,364,798-bp-long contig (PRJNA841434).

### 16S rRNA Sequencing and Phylogenetic Analysis

*S. marisflavi* BBL25 was identified at the species level with 16S rRNA sequencing by polymerase chain reaction amplification using primer 27F. Partial sequences were obtained from Cosmo Genetech (Korea) and compared to those in the National Center for Biotechnology Information (NCBI) GenBank database (https://blast.ncbi.nlm.nih.gov/Blast.cgi) using BLASTN [25]. Sequence homology assay results showed that the *S. marisflavi* strain BBL25 was 98.02% similar to the *S. marisflavi* strain 4-Sj-4-4-5-M (KJ009528.1) (Fig. S1).

### Plasmid Construction and Transformation of *S. oneidensis* MR-1

The strains and plasmids used in this study are listed in Table 1. Enzymes were purchased from New England Biolabs (USA). *Escherichia coli* DH5α was used for the construction of a recombinant vector and grown in LB medium containing kanamycin (50 μg/ml). The recombinant vector was constructed to determine the effects of the *glk* gene from *S. marisflavi* BBL25. *Shewanella oneidensis* MR-1 was cultivated overnight in 1 ml of LB medium and subsequently centrifuged (4°C, 3,700 ×*g* for 10 min). The pellets were washed twice and resuspended in a cooled 10% glycerol solution. The recombinant vector (pBBR1MCS2-*glk*) was added to resuspended cell pellets and subsequently electroporated using a 0.2 cm cuvette and a BioRad GenePulser (Bio-Rad, USA), followed by recovery in the LB medium at 30°C for 2 h [26]. As a selective marker, the engineered strain was selected on the LB medium containing kanamycin (50 μg/ml). For cell preparation, the strain was cultivated in LB medium at 30°C. To compare cell growth and glucose consumption, the engineered or wild-type *S. oneidensis* MR-1 strain was cultured in M9 minimal media containing 0.1% yeast extract and 1% glucose. After 24 h of culture, cell growth and glucose consumption of both strains were compared.

### Growth Conditions and Media Composition

All chemicals used for media preparation in this study were purchased from BD Difco Laboratories (Becton-Dickinson, USA). *S. marisflavi* BBL25 was cultured at 30°C in LB or M9 minimal media [Na_2_HPO_4_•7H_2_O 64 g/l, KH_2_PO_4_, 15 g/l NaCl, 2.5 g/l, NH_4_Cl, 5.0 g/l]. The cultured cells were stored in 20% glycerol at −80°C as stock solutions for further use. A single colony from the LB agar plate was precultured in 5 ml of LB broth [19]. The precultured cells were washed with phosphate-buffered saline (pH 7) twice and resuspended in M9 minimal medium containing 0.1% (w/v) yeast extract [27]. These aerobic precultures were used as the inoculum (1%, v/v) for anaerobic growth experiments with 50 ml serum bottles containing 20 ml of M9 minimal media comprising 0.1% yeast extract supplemented with the experiment-specific electron donor and acceptor pair. Anaerobic cultures were grown in 20 ml of M9 minimal media with pyruvate (40 mM) as the electron donor and fumarate as the electron acceptor (20 mM) in 50 ml serum bottles sealed with butyl-rubber stoppers in an anaerobic chamber (Coy Laboratory Products Inc., USA) under an atmosphere containing 85% N_2_, 10% CO_2_, and 5% H2. Oxygen was removed from the media by flushing the serum bottles with N_2_ gas. The N_2_ gas was blown through the media for 10 min to maintain an anaerobic environment, and rubber stoppers were used to prevent biogas escape. All bottles were sealed with rubber stoppers and removed from the anaerobic chamber. Biohydrogen was produced at 30°C and 200 rpm in a shaking incubator for 48 h.

### Analytical Methods

Reagents for GC and high-performance liquid chromatography (HPLC) were obtained from Sigma-Aldrich (USA). To measure biohydrogen production, biogas analysis was conducted using gas chromatography, which is described as follows: the volume of biogas released by each strain under each parameter was measured using a glass syringe (Sigma-Aldrich) and compared. In addition, 0.2 ml of biogas was collected from the headspace and analyzed to validate the biohydrogen content in the total biogas via gas chromatography (YL6500 GC; YoungIn Chromass, Korea) using a thermal conductivity detector with a 3.66 m × 3.18 mm × 2 mm Porapak N packed column (Agilent Technologies, USA) and N_2_ as the carrier gas. The temperatures of the inlet, oven, and GC system detector were set at 150, 80, and 150°C, respectively. The analysis time was 2 min, and a hydrogen peak was identified at approximately 0.8 min. The hydrogen concentration in all experiments was quantified in comparison with 0.2 ml of standard hydrogen gas (Scotty Gases; VWR Scientific, USA) [28]. To determine carbon source consumption, 0.2 ml culture medium was extracted from the anaerobic serum bottle using a 5 mL syringe (Korea Vaccine, Korea), vortexed for 5 s, and centrifuged at 13,000 ×*g* for 10 min [29]. To measure the residual carbon source, the supernatant (100 μl) was diluted with 900 μl of HPLC-grade distilled water (Thermo Fisher Scientific, USA) and filtered through a polyvinylidene fluoride membrane syringe (13 mm, CHROMDISC, Korea) with a pore size of 0.45 μm [30]. The residual carbon source concentration was measured by HPLC with a refractive index detector (Flexar HPLC System; Perkin Elmer, USA). The column was a 300 mm × 7.8 mm Aminex HPX-87H ion exclusion column (Bio-Rad). The mobile phase comprised 0.008 N sulfuric acid (H_2_SO_4_), and the experiment was performed at a flow rate of 0.6 ml/min for 40 min [31].

## Results

### Genomic Sequencing of *S. marisflavi* BBL25 and Identification of Associated Electron Transport Genes

**Fig. 1:**
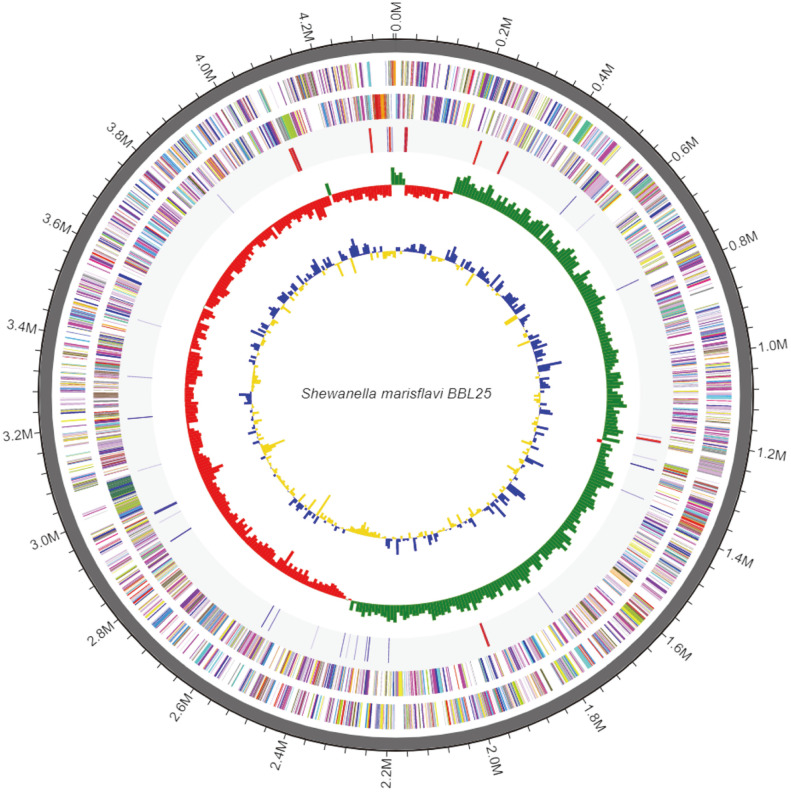
Whole-genome sequence map of *Shewanella marisflavi* BBL25.

*S. marisflavi* BBL25 produces electricity from lignocellulosic biomass and various carbon sources [32]. Compared with *S. oneidensis* MR-1, it is more efficient in utilizing carbon sources. In addition, *S. marisflavi* BBL25 produced polyhydroxybutyrate (PHB) using pLW487 containing PHB synthetic genes, suggesting it could potentially be used as a host for biochemical production [22]. As a result, to obtain genetic information on *S. marisflavi* BBL25, genome sequencing was performed. Whole-genome sequencing data of *S. marisflavi* BBL25 indicated that its genome was 4,364,798 bp long, with a 49.9% guanine-cytosine content and 3,845 open reading frames (ORFs) (Fig. 1, Table 2). Compared to the whole genomes of other *Shewanella* species, *S. marisflavi* BBL25 had a relatively small number of genes and genome size [33-39]. However, the G-C content was above average compared to that of other *Shewanella* species (Table 3). Functional categories were analyzed based on *S. marisflavi* BBL25 orthogonal group clusters (Table 4). A total of 7.20% of all genes were related to amino acid transport and metabolism, and 6.50% of genes were related to energy production and conversion. In other *Shewanella* strains, similarly high compositions of related genes were not observed [40].

The genes related to the *S. marisflavi* BBL25 electron transport system are shown in Table 5. We found two types of *mtrA* and *mtrB* and one type of *cymA* and *mrtC* genes in *S. marisflavi* BBL25. Two types of *mtrA* and *mtrB* genes suggest the possibility of higher electricity generation [41]. The primary periplasmic component encoded by the *mtrA* gene is used by MR-1 to reduce iron (III) and flavins [42]. The *mtrB* gene is known to be associated with the Mtr respiratory pathway [43]. The product of the *mtrC* gene has been purified and demonstrated to exchange electrons with each other and various forms of iron (III), flavins, and electrodes [43]. The *cymA* gene is a cytoplasmic membrane-anchored tetraheme c-type cytochrome that is capable of receiving electrons from the menaquinone pool [21].

### Identification of *S. marisflavi* BBL25 Biohydrogen-Producing Systems

**Fig. 2:**
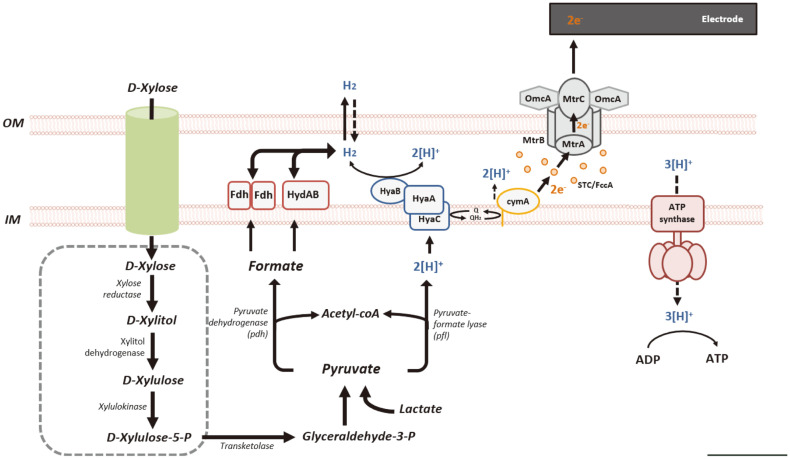
Schematic diagram of hydrogen production from xylose and electron transport system in *Shewanella marisflavi* BBL25.

**Fig. 3:**
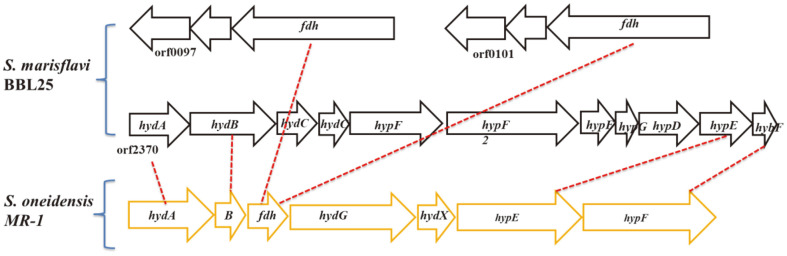
Comparison of hydrogen-producing genes in *Shewanella marisflavi* BBL25 and *S. oneidensis* MR-1.

Based on genome information and a deep search of metabolic genes, we assigned the metabolic pathway of xylose to pyruvate (Fig. 2). We also discovered hydrogen production-related gene clusters, suggesting the possibility of hydrogen production in *S. marisflavi* BBL25 (Table 6). Formate dehydrogenase plays a dominant role in the formate-driven hydrogen production [18] and serves as an important energy source for microorganisms during aerobic and anaerobic respiration. Oxidation of formate results in the generation of a pair of electrons, protons, and carbon dioxide molecules and is generally performed by the formate dehydrogenase complex of a cytoplasmic membrane [44]. Oxidation occurs in the periplasm to avoid cytoplasm acidification, and electrons are transferred via the formate dehydrogenase complex to menaquinone, along with protons from the cytoplasmic side of the inner membrane [44]. We found two formate dehydrogenases that played a critical role in hydrogen production in *Shewanella* species, in contrast to *S. oneidensis* MR-1 [13]. Based on this, we drew a schematic diagram of hydrogen production from xylose and an electron transport system (Fig. 2). In addition, we provided a comparative schematic diagram of the hydrogen-producing genes of *S. oneidensis* MR-1 and *S. marisflavi* BBL25 (Fig. 3). *Shewanella oneidensis* MR-1 genome information was obtained from the Kyoto Encyclopedia of Genes and Genomes (KEGG) [45].

### Production of Biohydrogen by *S. marisflavi* BBL25 with Various Carbon Sources

**Fig. 4:**
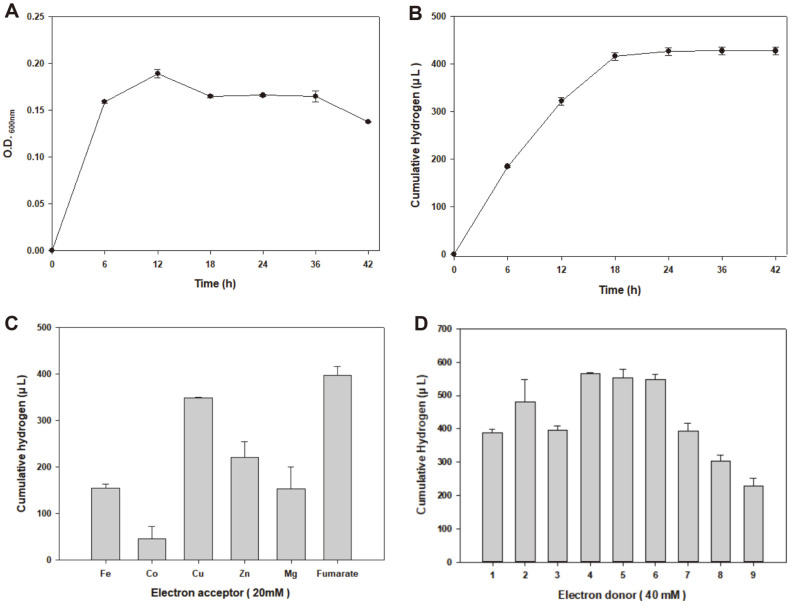
Confirmation of hydrogen production in *Shewanella marisflavi* BBL25 by comparing parameters affecting hydrogen production. (**A**) Growth of *S. marisflavi* BBL25. (**B**) Cumulative hydrogen production of *S. marisflavi* BBL25. (**C**) Comparison of hydrogen production by 20 mM of electron acceptor in S.marisflavi BBL25. (**D**) Comparison of hydrogen production by 40 mM of electron in S.marisflavi BBL25 (1 glucose, 2 fructose, 3 sucrose, 4 xylose, 5 glycerol, 6 galactose, 7 lactose, 8 pyruvate, 9 lactate).

*Shewanella* species have been recognized as potential biohydrogen producers owing to their ability to produce hydrogen from pyruvate under anaerobic stationary-phase conditions in the absence of an external electron acceptor [19]. Under anaerobic conditions, *S. oneidensis* MR-1 can oxidize lactate, pyruvate, formate, hydrogen, and some amino acids for respiration and may form a productive partnership with fermentative microorganisms [46]. In examining whether *S. marisflavi* BBL25 can produce hydrogen under the same conditions as those of *S. oneidensis* MR-1, we assumed that *S. marisflavi* BBL25 produces biohydrogen by utilizing pyruvate and grows under anaerobic conditions in the presence of external electron acceptor, fumarate. *S. marisflavi* BBL25 could produce biohydrogen and grow under the aforementioned conditions. The highest optical density (OD) (0.1890 ± 0.003) was recorded at 600 nm after 12 h of culture (Fig. 4A). However, the highest amount of biohydrogen (427.58± 5.85 μl) was recorded at 36 h after culture (Fig. 4B). Based on this, *S. marisflavi* BBL25 was capable of hydrogen production and growth under anaerobic conditions, and comparisons between influencing factors such as electron donors and acceptors were performed because biohydrogen production in *Shewanella* species is affected by electron donors and acceptors [47]. The electron acceptors used by *S. oneidensis* MR-1 include soluble organic compounds, such as fumarate, insoluble extracellular metal oxides, and electrodes [46]. We found that hydrogen production was the highest when sodium fumarate was used as an electron acceptor and the lowest when Co^2+^ was used (Fig. 4C). The *Shewanella* species obtain energy for growth during anaerobic respiration, in which various organic acids or cathode hydrogen act as electron donors [48]. To determine the effect of the electron donor, 40 mM pyruvate, lactate, glucose, fructose, sucrose, xylose, glycerol, galactose, and lactose were used as electron donors. Fumarate was used as the electron acceptor, and various electron donors were examined at an amount of 40 mM. *S. marisflavi* BBL25 produced the highest amount of hydrogen from xylose (566.55 ± 0.45 μl), followed by glycerol and galactose (Fig. 4D).

**Fig. 5:**
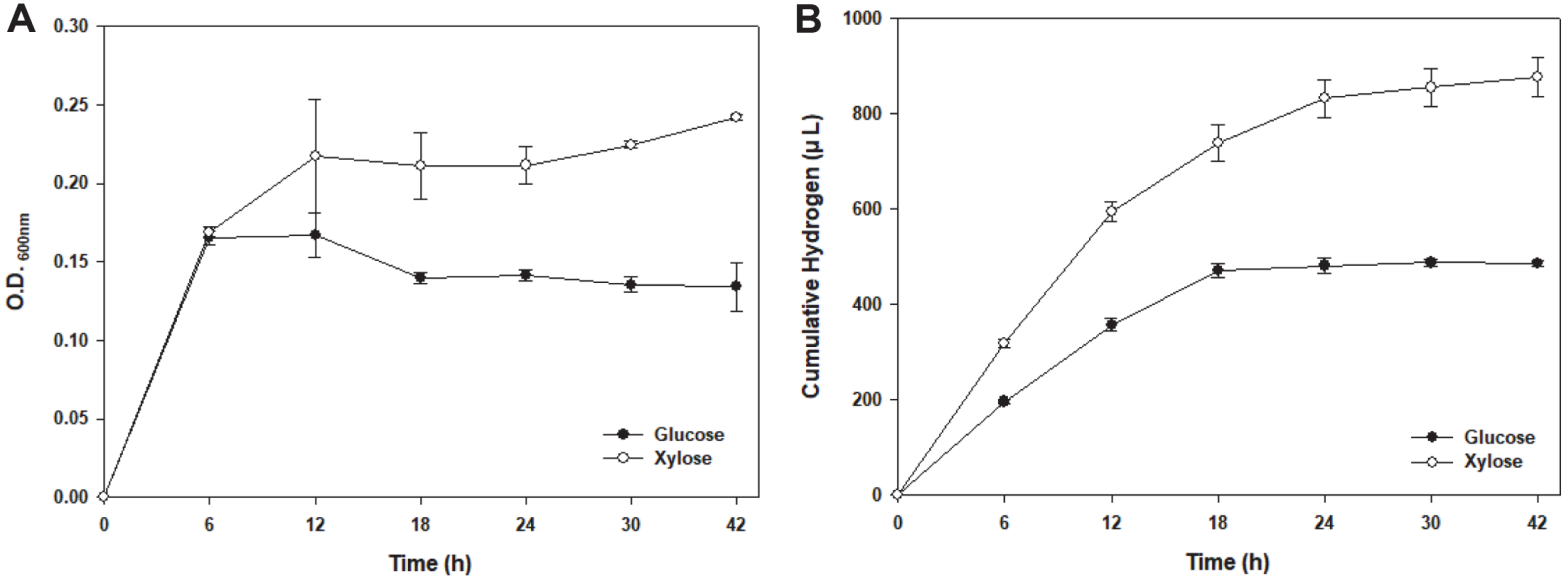
Time-dependent monitoring of *Shewanella marisflavi* BBL25 biohydrogen production using glucose and xylose. (**A**) Growth of *S. marisflavi* BBL25 using glucose and xylose (**B**) Cumulative *S. marisflavi* BBL25 hydrogen production using glucose and xylose.

The time-dependent production of biohydrogen by *S. marisflavi* BBL25 was determined using glucose and xylose. In this experiment, S. marisflavi BBL25 was cultured in M9 minimal media containing 20 mM of sodium fumarate and 60 mM of xylose or glucose. As a result, in the case of using glucose as an electron donor, *S. marisflavi* BBL25 showed the highest OD of 0.167 ± 0.01 at 600 nm after 12 h of culture (Fig. 5B). The highest cumulative hydrogen volume (487.08 ± 4.44 μl) was observed after 42 h of culture (Fig. 5A). With xylose as an electron donor, *S. marisflavi* BBL25 showed the highest OD (0.242 ± 0.01 at 600 nm) and the highest cumulative hydrogen volume (877.43 ± 28.53 μl at 600 nm) after 42 h of culture. The results of growth and hydrogen production from xylose are shown in Figs. 5A and 5B. These results showed that *S. marisflavi* BBL25 can produce more hydrogen from xylose than glucose and can use multiple carbon sources.

### Comparison of Sugar-Utilizing Genes and Application of *glk* in *S. oneidensis* MR-1

*S. marisflavi* BBL25 can grow using various carbon sources and generate electricity, especially from C6 biosugars [22]. We analyzed carbon source uptake and performed a systematic analysis of *S. marisflavi* BBL25. *Shewanella marisflavi* BBL25 contains D-glucose PTS permease (orf_01478, orf_01477), IIA (orf_02257), glucokinase (orf_02722), glucose-6-phosphate isomerase (orf_03084), fructose-bisphosphatase (orf_03413), aldolase (orf_00197), phosphoglycerate kinase (orf_00196), and pyruvate kinase (orf_02062) (Table 7). Unlike *S. oneidensis* MR-1, *S. marisflavi* BBL25 contains a glucokinase-encoding gene (*glk*) in the genome. In addition, the metabolic pathway of glucose utilization of *S. marisflavi* BBL25 is well established due to whole-genome sequencing. Hence, there is a possibility that genes related to the glucose utilization by *S. marisflavi* BBL25 can be used for the same purpose in other strains that cannot utilize glucose. For xylose utilization, we identified a xylose-utilizing system in *S. marisflavi* BBL25. It comprised xylose reductase (orf_00894), xylitol dehydrogenase (orf_03529), xylulokinase (orf_02525), and transketolase (orf_00194) (Table 7). In the *S. marisflavi* BBL25 xylose utilization system, genes encoding xylose isomerase (*xylA*) and xylulokinase (*xylB*) were not found [49]. Moreover, we could not assign and specify xylose-specific transport systems. Considering that xylose availability has previously been shown, its presence can be inferred. Finally, an *N*-acetylglucosamine uptake system in *S. marisflavi* BBL25, a well-known carbon source for *S. oneidensis* MR-1 [50], was identified. *S. marisflavi* BBL25 comprises an *N*-acetylglucosamine symporter (orf_03052), *N*-acetylglucosamine permease (orf_02284), *N*-acetylglucosamine kinase (orf_02722), *N*-acetylglucosamine-6-phosphate deacetylase (orf_03054), and glucosamine-6-phosphate deaminase (orf_03370). Overall, *Shewanella oneidensis* MR-1 cannot catabolize glucose because the glucokinase-encoding gene (*glk*) is not present in its genome. Moreover, xylose cannot be oxidized to xylulose because xylitol dehydrogenase (XDH) encoded by the gene *xyl2* is absent [51, 52].

**Fig. 6:**
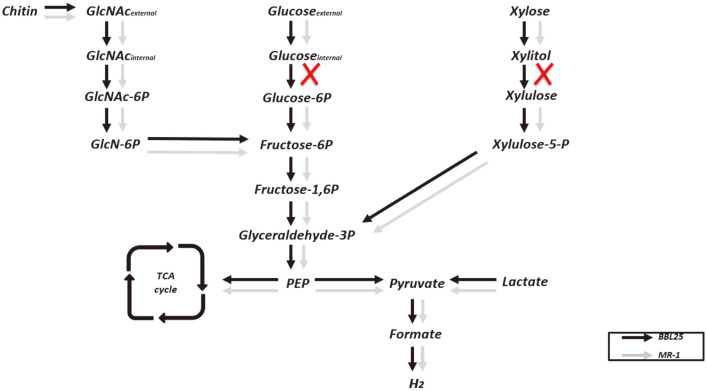
Comparison of sugar metabolism-associated genes in *S. marisflavi* BBL25 and *S. oneidensis* MR-1.

**Fig. 7:**
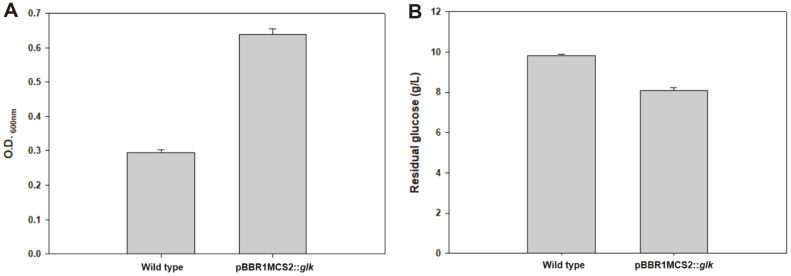
Comparison of glucose utilization in the wild-type *Shewanella oneidensis* MR-1 and engineered *S. oneidensis* MR-1 (pBBR1MCS2::*glk*). Growth of engineered *S. oneidensis* and wild-type *S. oneidensis* MR-1. (**B**) Residual glucose of engineered *S. oneidensis* and wild-type *S. oneidensis* MR-1.

Genes related to glucose and xylose utilization were found in *S. marisflavi* BBL25, suggesting that glucose and xylose utilization in *S. marisflavi* BBL25 may be more valuable than in *S. oneidensis* MR-1 (Fig. 6). Although *S. marisflavi* BBL25 produced more hydrogen than glucose when it used xylose, it is important in enabling glucose utilization for biohydrogen production because most microbial biohydrogen production systems focus on hexose glucose [53, 54]. To determine if glucose utilization in *S. oneidensis* MR-1 could be possible due to the absence of *S. marisflavi* BBL25 gene *glk* in *S. oneidensis* MR-1 for utilization of glucose, the recombinant vector (pBBR1MCS2::*glk*) was introduced into *S. oneidensis* MR-1. We then compared the growth and glucose consumption of this strain and found that the engineered *S. oneidensis* MR-1 had a higher OD of 0.639 ± 0.01 at 600 nm and consumed 18.3%more glucose than the wild type. In contrast, wild-type MR-1 showed a lower OD of 0.29 ± 0.01 at 600 nm, and carbon source consumption was not observed (Fig. 7).

## Discussion

In this study, we previously found that *S. marisflavi* BBL25 can utilize various carbon sources and produce electricity. However, the mechanism involved remained unclear. Using genome sequencing, we demonstrated the existence of genes related to carbon source uptake, utilization systems, and electron transfer systems. We also identified the pathways for the biohydrogen-producing system in *S. marisflavi* BBL25. After whole-genome sequencing of this strain, we attempted to identify and confirm its potential as a hydrogen producer. In comparison to hydrogen production in *S. oneidensis* MR-1, *S. marisflavi* BBL25 had more formate dehydrogenase, which indicated that BBL25 had greater potential as a hydrogen producer. This was confirmed by hydrogen production experiments under anaerobic conditions. Moreover, by introducing the *glk* gene from *S. marisflavi* BBL25 into *S. oneidensis* MR-1, we observed glucose utilization and growth using glucose in *S. oneidensis* MR-1. Based on these findings, we verified that the glucose utilization system of *S. marisflavi* BBL25 worked, and the possibility of improving *S. oneidensis* MR-1 using a carbon source-utilizing gene from *S. marisflavi* BBL25 was confirmed. Thus, our study demonstrated various carbon sources and biohydrogen production systems, which offset the shortcomings of the existing representative strains. Furthermore, the whole-genome data generated in this study indicated the possibility of engineering this strain for higher electricity generation, hydrogen production, and carbon source utilization in other *Shewanella* species.

## Supplemental Materials

Supplementary data for this paper are available on-line only at http://jmb.or.kr.

## Figures and Tables

**Table 1:** Bacterial strains and plasmids used in this study.

Plasmid or strain	Description	Source
Plasmid		
pBBR1MCS2	Km^r^, pBBR1 replicon, mob^+^	Lab stock
pBBR1MCS2::*glk*	pBBR1MCS2 carrying *glk* gene from *S. marisflavi* BBL25	Present study
Strain		
*E. coli* DH5α	*dlacZ* Δ*M15* Δ(*lacZYA-argF*) *U169 recA1 endA1* *hsdR17(rK-mK* +) *supE44 thi-1 gyrA96 relA*	Lab stock
*S. marisflavi* BBL25	Wild-type strain	Lab stock
*S. oneidensis* MR-1	Wild-type strain	Lab stock

**Table 2:** Whole genome characteristics of *Shewanella marisflavi* BBL25^a^.

Attributes	Values
Genome size (bp)	4,364,798
G+C ratio (%)	49.9
Number of ORFs	3,845
Number of rRNA genes	32
Number of tRNA genes	103

^a^ORFs; open reading frames; tRNA, transfer RNA; G+C, guanine+cytosine ratio

**Table 3:** Comparison of genome characteristics between *Shewanella marisflavi* BBL25 and other *Shewanella* species.

Species	Genome size (Mb)	GC^a^contents (%)	Gene count	Reference
*S. oneidensis* MR-1	4.97	46.0	4758	[33]
*S. psychrophile* WP2	6.35	44.3	5503	[34]
*S. benthica* DB21MT-2	4.35	45.9	4457	[35]
*S. algae* YHL	4.85	53.0	4212	[36]
*S. piezotolerans* WP3	5.40	43.3	4933	[37]
*S*. sp. Strain TH2012	4.86	54.8	4176	[38]
*S. marisflavi* ECSMB1410	4.34	49.9	3891	[39]
*S. marisflavi* BBL25	4.36	49.9	3845	Present study

^a^GC, guanine and cytosine

**Table 4:** Functional categories based on clusters of orthologous groups (COG) of *Shewanella marisflavi* BBL25.

Description	COG	Number of genes	%
Cell cycle control, cell division, chromosome partitioning	D	33	0.91%
Cell wall/membrane/envelope biogenesis	M	177	4.90%
Cell motility	N	57	1.58%
Post-translational modification, protein turnover, chaperones	O	169	4.68%
Signal transduction mechanisms	T	218	6.03%
Intracellular trafficking, secretion, and vesicular transport	U	86	2.38%
Defense mechanisms	V	44	1.22%
RNA processing and modification	A	1	0.0028%
Chromatin structure and dynamics	B	1	0.0028%
Translation, ribosomal structure and biogenesis	J	187	5.18%
Transcription	K	190	5.25%
Replication, recombination and repair	L	163	4.51%
Energy production and conversion	C	235	6.50%
Amino acid transport and metabolism	E	260	7.20%
Nucleotide transport and metabolism	F	71	1.97%
Carbohydrate transport and metabolism	G	109	3.02%
Coenzyme transport and metabolism	H	128	3.54%
Lipid transport and metabolism	I	99	2.74%
Inorganic ion transport and metabolism	P	181	5.01%
Secondary metabolite biosynthesis, transport and catabolism	O	43	1.20%
General function prediction only	R	0	0%
Function unknown	S	1161	32.1%
Total		3613	100%

**Table 5:** Electron transport system-related genes of *Shewanella marisflavi* BBL25^a^.

Sequence name	Sequence description	Sequence length	Gene ontologies	EggNOG ID	EggNOD category	Direction
orf_01585	*mtrC* BLAST deca-heme cytochrome c	2004		ENOG410YDEX	C	Reverse
orf_01584	*mtrA* BLAST cytochrome C family protein	990		ENOG410XNV6	G	Reverse
orf_01583	*mtrB* BLAST Outer membrane protein, MtrB	2100		ENOG4111RQK	M	Reverse
orf_03812	*cymA* BLAST Denitrification system component NirT	564	0016021, 0005886, 0020037, 0046872, 0019333, 0055114	COG3005	C	Normal
orf_01591	*mtrA* BLAST low ident. cytochrome C family protein	966		ENOG410XNV6	C	Reverse
orf_01590	*mtrB* BLAST low ident. Outer membrane protein, MtrB	2,112		ENOG4111RQK	M	Reverse

^a^ORF, open reading frame

**Table 6:** Biohydrogen-producing system-related genes of *Shewanella marisflavi* BBL25^a^.

Sequence name	Sequence description	Sequence length	Gene ontologies	EggNOG ID	EggNOD category	Direction
orf_02370	Hydrogen:quinone oxidoreductase(HydA)	1137	0009375, 0042597, 0051538, 0051539, 0047806, 0008901, 0046872	COG1740	C	Reverse
orf_02371	Hydrogen:quinone oxidoreductase (HydB)	1707	0005737, 0008901, 0047985, 0016151	COG0374	C	Reverse
orf_02372	Quinone-reactive Ni/Fe-hydrogenase B-type cytochrome subunit	672	0016021, 0005886, 0009055, 0005506, 0022904, 0006810	COG1969	C	Reverse
orf_02373	Protein HydD	564	0004190, 0008047	COG0680	C	Reverse
orf_02374	Ni-Fe hydrogenase	1758		EGNOG41 11KJA	S	Reverse
orf_02375	Carbamoyltransferase HypF2	2370	0016743, 0003725, 0008270, 0046944	COG0068	O	Reverse
orf_02376	Hydrogenase/urease nickel incorporation protein HypB	780	0016151, 0006461	COG0378	KO	Reverse
orf_02377	Hydrogenase expression/ formation protein HypC	249		COG0298	O	Reverse
orf_02378	Hydrogenase isoenzyme formation protein HypD	1116	0051539, 0070025, 0005506, 0006464, 0051604	COG0409	O	Reverse
orf_02379	Hydrogenase expression/ formation protein HypE	1017		COG0309	O	Reverse
orf_02380	Hydrogenase/urease nickel incorporation protein HypF	351	0016151, 0006464	COG0375	O	Reverse
orf_00103	Formate dehydrogenase	2850	0051539, 0009055, 0046872, 0016491	COG0243	C	Normal
orf_00102	Formate dehydrogenase ironsulfur subunit	591	0051539, 0009055, 0046872, 0055114	COG0437	C	Normal
orf_00101	Formate dehydrogenase-O, gamma subunit	1008		COC2864	C	Normal
orf_00099	Formate dehydrogenase	2856	0051539, 0009055, 0046872, 0016491	COG0243	C	Normal
orf_00098	Formate dehydrogenase ironsulfur subunit	570	0051539, 0009055, 0046872, 0055114	COG0437	C	Normal
orf_00097	ormate dehydrogenase -O, gamma subunit	993		COG2864	C	Normal

^a^ORF, open reading frame

**Table 7:** Carbon sources (glucose, xylose, and N-acetylglucosamine) utilizing system-related genes of *Shewanella marisflavi* BBL25^a^.

Sequence name	Sequence description	Sequence length	Gene ontologies	EggNOG ID	EggNOD category
orf_01477	Protein-N(pi)-phosphohistidine-- D-glucose phosphotransferase	405	0016021, 0005886, 0005355, 0016301, 0008982, 0009401	COG1263	G
orf_01478	Protein-N(pi)-phosphohistidine-- D-glucose phosphotransferase	1059	0016021, 0005886, 0005355, 0016301, 0008982, 0009401	COG1263	G
orf_02257	Glucose-specific phosphotransferase enzyme IIA component	510	0005737, 0016301, 0046872, 0009401	COG2190	G
orf_02722	Glucokinase	891	0005829, 0005524, 0008865, 0004396, 0046835, 0019318	COG1940	G
orf_03084	Glucose-6-phosphate isomerase	1638	0005737, 0004347, 0006094, 0006096	COG1066	G
orf_03413	Fructose-bisphosphatase	990	0005737, 0042132, 0000287, 0006094	COG0158	G
orf_00917	Tagatose-bisphosphate aldolase	1065	0004332, 0008270, 0006096, 0019253	COG0191	G
orf_00916	Phosphoglycerate kinase	1176	0005737, 0005524, 0004618, 0006096	COG0126	G
orf_02062	Pyruvate kinase	1440	0005524, 0016301, 0000287, 0030955, 0004743	COG0469	G
orf_00894	Xylose reductase	1041	0005829, 0004033, 0034198	COG0667	C
orf_03529	Xylitol dehydrogenase	1065	0048037, 0016491, 0008270	COG0604	C
orf_02525	Xylulokinase	1548	0005524, 0004370, 0019563, 0006071, 0006072	COG1070	G
orf_00914	Transketolase	1995	0005829, 0030145, 0030976, 0004802, 0009052	COG0021	G
orf_03052	N-acetylglucosamine symporter	1299	0016021, 0005886, 0015518, 0015535, 0015517, 0015751, 0006004, 0015756, 0015757	COG0738	G
orf_02284	N-acetylglucosamine permease	1230	0016021, 0005886, 0005215, 0055085	COG2233	F
orf_02722	N-acetylglucosamine kinase	891	0005829, 0005524, 0008865, 0004396, 0046835, 0019318	COG1940	G
orf_03054	N-acetylglucosamine-6-phosphate deacetylase	1131	0046872, 0008448, 0005975, 0006046, 0006044, 0019262, 0051289	COG1820	G
orf_03370	Glucosamine-6-phosphate deaminase	804	0004342, 0016787, 0005975, 0006044, 0019262	COG0363	G

^a^ORF, open reading frame
