# Supplementary material for: Unraveling Biohydrogen Production and Sugar Utilization Systems in the Electricigen Shewanella marisflavi BBL25
Source: J Microbiol Biotechnol. 2023 Feb 15;33(5):687–97. doi: 10.4014/jmb.2212.12024 (PMC10236175; doi:10.4014/jmb.2212.12024)
Supplement: Supplementary file 1 [file jmb-33-5-687-supple.pdf]

J. Microbiol. Biotechnol.
https://doi.org/10.4014/jmb.2212.12024

Supplementary Figures

Unraveling Biohydrogen Production and Sugar Utilization Systems in the Electricigen *Shewanella marisflavi* BBL25

Figure. 1. Phylogenetic tree of *Shewanella marisflavi* BBL25 based on 16s rRNA sequencing.

Figure. 2. Supplementary Fig. S2. Functional categories based on clusters of orthologous groups (COG) of *Shewanella marisflavi* BBL25/

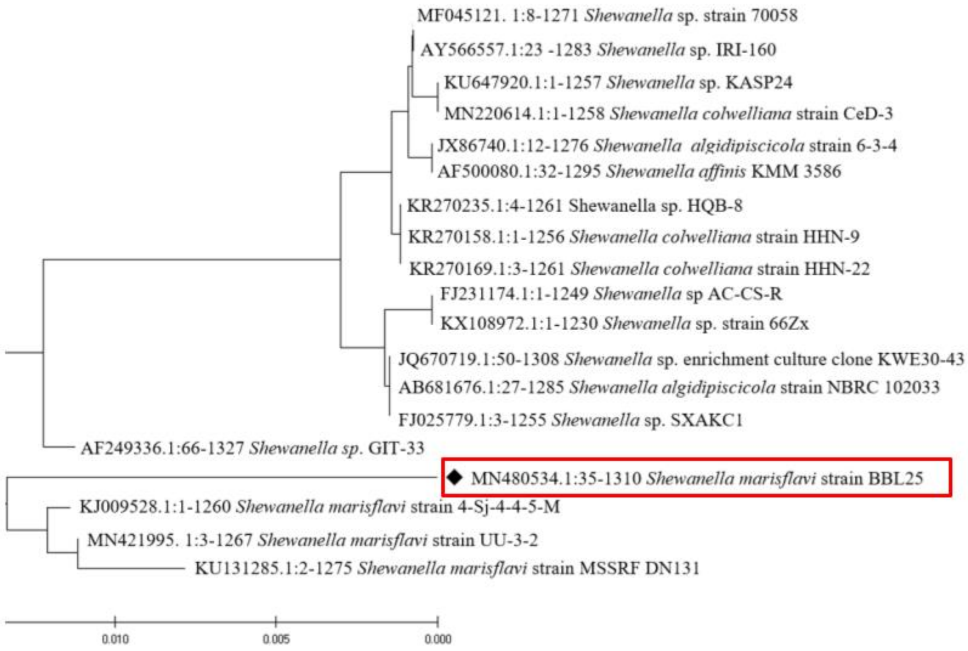

Supplementary Figure 1.

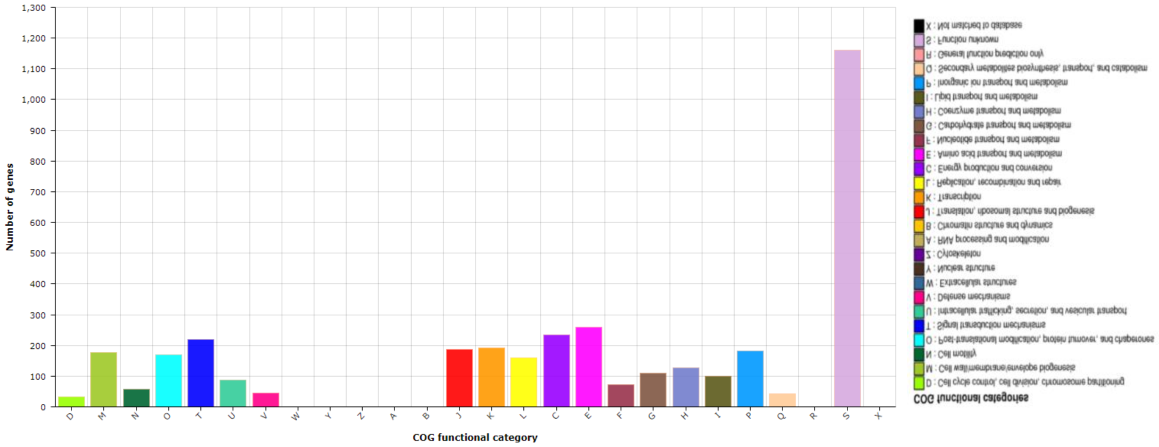

Supplementary Figure 2.
